# Supplementary material for: Learning to simulate high energy particle collisions from unlabeled data
Source: Sci Rep. 2022 May 9;12:7567. doi: 10.1038/s41598-022-10966-7 (PMC9085893; doi:10.1038/s41598-022-10966-7)
Supplement: Supplementary file 1 — Supplementary Information. [file 41598_2022_10966_MOESM1_ESM.pdf]

1 Supplementary Information

1.1 Supplementary Statistics

	z vs $\tilde{z}$		
	**W [GeV^2]**	**(χ^2_R, dof)**	**KS**
**Figure 3a** (p_y)	$1.34 \times 10^{+00}$	(50.583, 23)	1.61×10^{-02}
**Figure 3a** (p_z)	$1.59 \times 10^{+00}$	(1.325, 26)	4.90×10^{-03}
**Figure 3a** (E)	$1.29 \times 10^{+00}$	(8.814, 26)	1.47×10^{-02}
**Figure 5a**	$2.73 \times 10^{+01}$	(822.762, 39)	2.46×10^{-01}

Supplementary Table 1. Table showing $\mathcal{Z}$ space statistical test results for the $Z \to e^+e^-$ dataset. These tests were performed on the distributions in the referenced figures in the main text. W is the Wasserstein distance, χ^2_R is the reduced χ^2 and dof is the degrees-of-freedom, and KS is the value of the Kolmogorov-Smirnov statistical test. See the Evaluation section in the main text for detailed information about the calculations of these statistics.

	x vs $\tilde{x}$			x vs $\tilde{x}'$		
	**W [GeV^2]**	**(χ^2_R, dof)**	**KS**	**W [GeV^2]**	**(χ^2_R, dof)**	**KS**
**Figure 3b** (p_y)	4.22×10^{-01}	(1.391, 23)	3.48×10^{-03}	$1.05 \times 10^{+00}$	(37.560, 23)	1.22×10^{-02}
**Figure 3b** (p_z)	$3.71 \times 10^{+00}$	(1.523, 26)	1.03×10^{-02}	$9.53 \times 10^{+00}$	(4.775, 26)	7.49×10^{-03}
**Figure 3b** (E)	6.64×10^{-01}	(0.489, 26)	3.19×10^{-03}	$3.64 \times 10^{+00}$	(9.370, 26)	2.00×10^{-02}
**Figure 5b**	7.28×10^{-01}	(5.055, 39)	2.61×10^{-02}	7.15×10^{-01}	(12.821, 39)	3.14×10^{-02}

Supplementary Table 2. Table showing $\mathcal{X}$ space statistical test results for the $Z \to e^+e^-$ dataset. These tests were performed on the distributions in the referenced figures in the main text. W is the Wasserstein distance, χ^2_R is the reduced χ^2 and dof is the degrees-of-freedom, and KS is the value of the Kolmogorov-Smirnov statistical test. See the Evaluation section in the main text for detailed information about the calculations of these statistics.

	z vs $\tilde{z}$		
	**W [GeV^2]**	**(χ^2_R, dof)**	**KS**
**Figure 6a** (p_y)	$1.58 \times 10^{+01}$	(7.418, 49)	1.25×10^{-02}
**Figure 6a** (p_z)	$5.52 \times 10^{+01}$	(4.613, 55)	1.65×10^{-02}
**Figure 6a** (E)	$6.20 \times 10^{+01}$	(31.228, 31)	4.04×10^{-02}

Supplementary Table 3. Table showing $\mathcal{Z}$ space statistical test results for the semileptonic $t\bar{t}$ dataset. These tests were performed on the distributions in the referenced figures in the main text. W is the Wasserstein distance, χ^2_R is the reduced χ^2 and dof is the degrees-of-freedom, and KS is the value of the Kolmogorov-Smirnov statistical test. See the Evaluation section in the main text for detailed information about the calculations of these statistics.

	x vs $\tilde{x}$			x vs $\tilde{x}'$		
	**W [GeV^2]**	**(χ^2_R, dof)**	**KS**	**W [GeV^2]**	**(χ^2_R, dof)**	**KS**
**Figure 6b** (p_y)	$2.40 \times 10^{+01}$	(2.395, 49)	1.66×10^{-02}	$1.23 \times 10^{+02}$	(34.021, 49)	4.59×10^{-02}
**Figure 6b** (p_z)	$1.08 \times 10^{+02}$	(0.828, 55)	9.90×10^{-03}	$3.42 \times 10^{+02}$	(1.980, 55)	6.94×10^{-03}
**Figure 6b** (E)	$4.11 \times 10^{+01}$	(1.281, 30)	1.02×10^{-02}	$3.24 \times 10^{+02}$	(50.072, 30)	4.80×10^{-02}
**Figure 8a**	$1.60 \times 10^{+02}$	(1.192, 43)	7.63×10^{-03}	$1.03 \times 10^{+03}$	(54.598, 43)	1.03×10^{-01}
**Figure 8b**	9.30×10^{-01}	(3.974, 35)	1.66×10^{-02}	$1.04 \times 10^{+02}$	(68.392, 35)	1.09×10^{-01}
**Figure 8c**	$8.83 \times 10^{+00}$	(1.579, 30)	5.91×10^{-03}	$7.41 \times 10^{+01}$	(92.533, 30)	1.31×10^{-01}
**Figure 8d**	$2.21 \times 10^{+01}$	(2.455, 41)	1.72×10^{-02}	$1.11 \times 10^{+03}$	(160.712, 41)	2.35×10^{-01}

Supplementary Table 4. Table showing $\mathcal{X}$ space statistical test results for semileptonic $t\bar{t}$ dataset. These tests were performed on the distributions in the referenced figures in the main text. W is the Wasserstein distance, χ^2_R is the reduced χ^2 and dof is the degrees-of-freedom, and KS is the value of the Kolmogorov-Smirnov statistical test. See the Evaluation section in the main text for detailed information about the calculations of these statistics.

1.2 Supplementary Schematic Diagram of Network Model

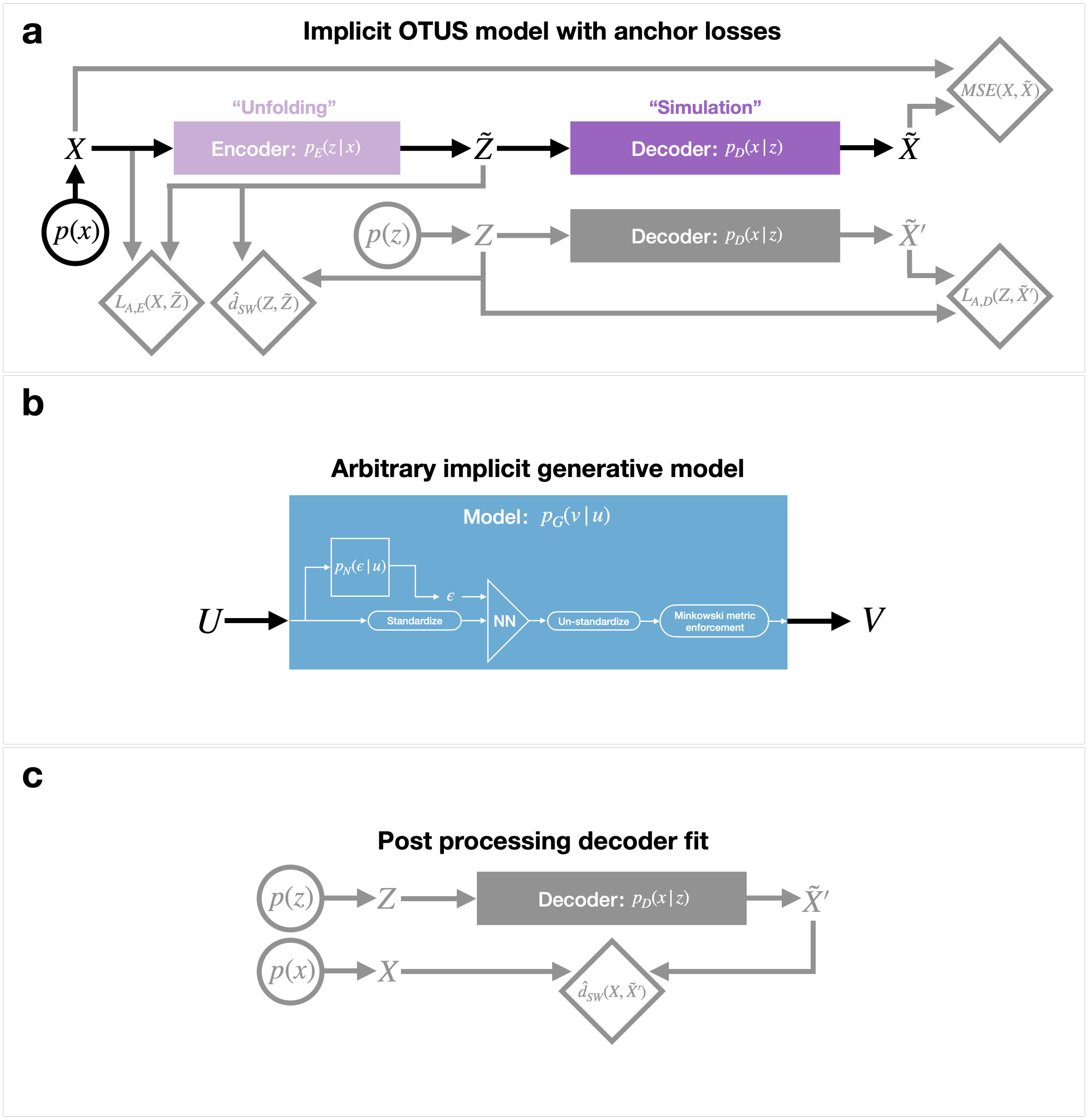

Supplementary Figure 1. Schematic diagrams of the network and loss structures used in this study for the base training strategy. a Diagram showing the full OTUS model where gray indicates information used in the calculation of losses only. **b** Diagram showing the internal structure present in both the encoder and decoder models. **c** Diagram showing the setup used for the post processing decoder network loss. See the text for more details.

1.3 Supplementary Ablation Study

In this section we show the results of an ablation study to demonstrate the effect of the various hyperparameters. As seen in our final loss function Equation (11),the main hyperparameters of our approach are the λ coefficient in front of the latent space loss, as well as the β_E and β_D coefficients weighing the anchor losses for the encoder and decoder, respectively. For the semileptonic $t\bar{t}$ study the only hyperparameter is λ, as the anchor loss is redundant with the choice of a ResNet [1] architecture (see Section 6.2.3). We performed ablations by retraining the models as in Section 6.3.3 but with different values of the hyperparameters on a grid, and comparing the results on validation data.

For studying the effect of λ, we reran both the $Z \to e^+e^-$ and the semileptonic $t\bar{t}$ studies with λ in {0.001, 0.01, 0.1, 1, 10, 100, 1000}, while keeping all other hyperparamters unchanged (specifically, in the $Z \to e^+e^-$ study we kept $\beta_E = \beta_D = 50$). For the effect of the anchor loss coefficients, we always assume that $\beta_E = \beta_D$ and define a shared hyperparameter $\beta := \beta_E = \beta_D$. We reran the $Z \to e^+e^-$ study with β in {0, 10, 20, 50, 100, 200}, while keeping $\lambda = 1$ as in the original experiment. We did not repeat this for the semileptonic $t\bar{t}$ study as it did not use an anchor loss.

We first consider how the hyperparameters on the anchor loss terms, $\beta_E = \beta_D$, affect performance. The anchor losses are direct constraints on the learned encoding and decoding mappings which are based on physical concerns. Namely, the anchor loss penalizes networks which would map electron/positron ($e^{\mp}$) information in $\mathcal{Z}$ to positron/electron ($e^{\pm}$) information in $\mathcal{X}$, and vice versa. We impose this constraint because we know that misidentification of charge in the process of data reconstruction is extremely rare in particle experiments. Therefore, for our simulation to be physical, it should not make these unphysical inversions. Unsurprisingly, without this constraint we can see that these inversions can occur during training (see Supplementary Figure 2). On the other hand, if the values of $\beta_E = \beta_D$ are too high we observe unphysical behavior. This is likely due to the fact that the anchor loss is only a proxy for enforcing charge conservation.

We next consider the hyperparameter λ which is present in both case studies. The behavior of λ has theoretical motivations. The WAE method aims to minimize $W_c(p(x), p_D(x))$ by converting its calculation into a constrained optimization problem. It was shown [2] that $W_c(p(x), p_D(x)) = \inf_{p_E(z|x):p_E(z)=p(z)} \mathbb{E}[c(X, D(Z))]$ for a deterministic decoder $p_D(x|z) = \delta_{D(z)}(x)$, [1]. Namely, we need to minimize a reconstruction error over all probabilistic encoders, $p_E(z|x)$, satisfying the latent-space matching condition, $p(z) \stackrel{!}{=} p_E(z) =: \int_x p_E(z|x)p(x)dx$. To make the constrained optimization computationally tractable, the WAE method only softly enforces this constraint via a penalty term $\lambda d_z(p(z), p_E(z))$, and considers minimizing the surrogate penalty loss $\mathbb{E}_{p(x)p_E(z|x)p_D(\tilde{x}|z)}[c(x,\tilde{x})] + \lambda d_z(p(z), p_E(z))$ instead.

By standard results on penalty methods [3], for a fixed decoder, $p_D(x|z)$, globally minimizing the penalty loss with respect to the encoder $p_E(z|x)$ results in a lower bound on $W_c(p(x), p_D(x))$, and solving a sequence of such penalized problems while annealing λ towards infinity results in the exact $W_c(p(x), p_D(x))$. However, when training a WAE, it is expensive to repeat this inner optimization procedure after every decoder update, so in practice both the encoder and decoder are optimized jointly on a penalty loss, keeping λ fixed throughout the entire training [2].

While the theoretical guarantees of the penalty method no longer applies to the joint Stochastic Gradient Descent training procedure used in practice, it does suggest that λ should be set to be as large as possible (and perhaps annealed during training) to better enforce the latent space matching, and consequently offer a better approximation of the ideal objective $W_c(p(x), p_D(x))$. Indeed, recently it was proven [4] that perfect latent space matching $p_E(z) == p(z)$ is a necessary condition for $W(p(x), p_D(x)) = 0$.

Overall, our ablation experiments confirmed this notion and showed that when λ is too small and thus the penalty on latent space matching too week, neither the encoder or the decoder's marginal distribution $(p_E(z), p_D(x))$ could capture the ground truth $p(z)$ or $p(x)$ well, despite minimal reconstruction error. We see this behavior in both test cases, however we note that in the semileptonic $t\bar{t}$ the behavior is somewhat less dramatic because of the heavy initial bias towards an identity mapping due to the ResNet [1] architecture (see Supplementary Figure 3).

---

[1] We can show that more generally, for a stochastic decoder, we have an upper bound $W_c(p(x), p_D(x)) \leq \inf_{p_E(z|x):p_E(z)=p(z)} \mathbb{E}_{p(x)p_E(z|x)p_D(\tilde{x}|z)}[c(X, \tilde{X})]$

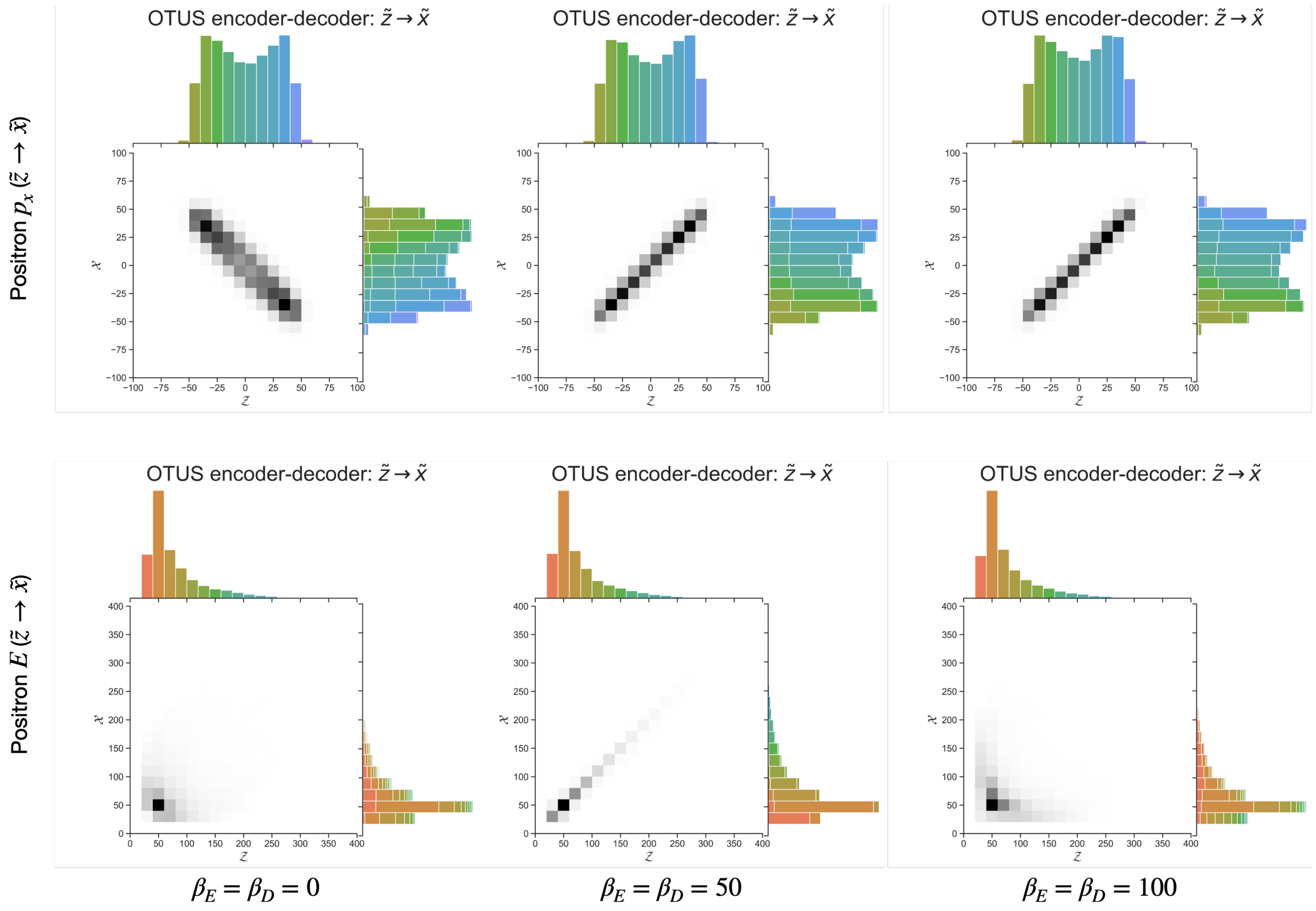

Supplementary Figure 2. Results of anchor loss ablation study in the $Z \to e^+e^-$ study. For $\beta_E = \beta_D = 0$ we can see that unphysical transformations can arise. In p_x, negative values in $\mathcal{Z}$ are being mapped to positive values in $\mathcal{X}$. This is a result of $e^{\pm}$ information being swapped in the learned transformation. For $\beta_E = \beta_D = 50$, this effect goes away; we also see more physical behavior in E as well. For $\beta_E = \beta_D = 100$, we observe that high values of β_E and β_D inadvertently encourage unphysical behavior in E. This is likely due to the fact that the anchor loss is only a proxy for enforcing charge conservation.

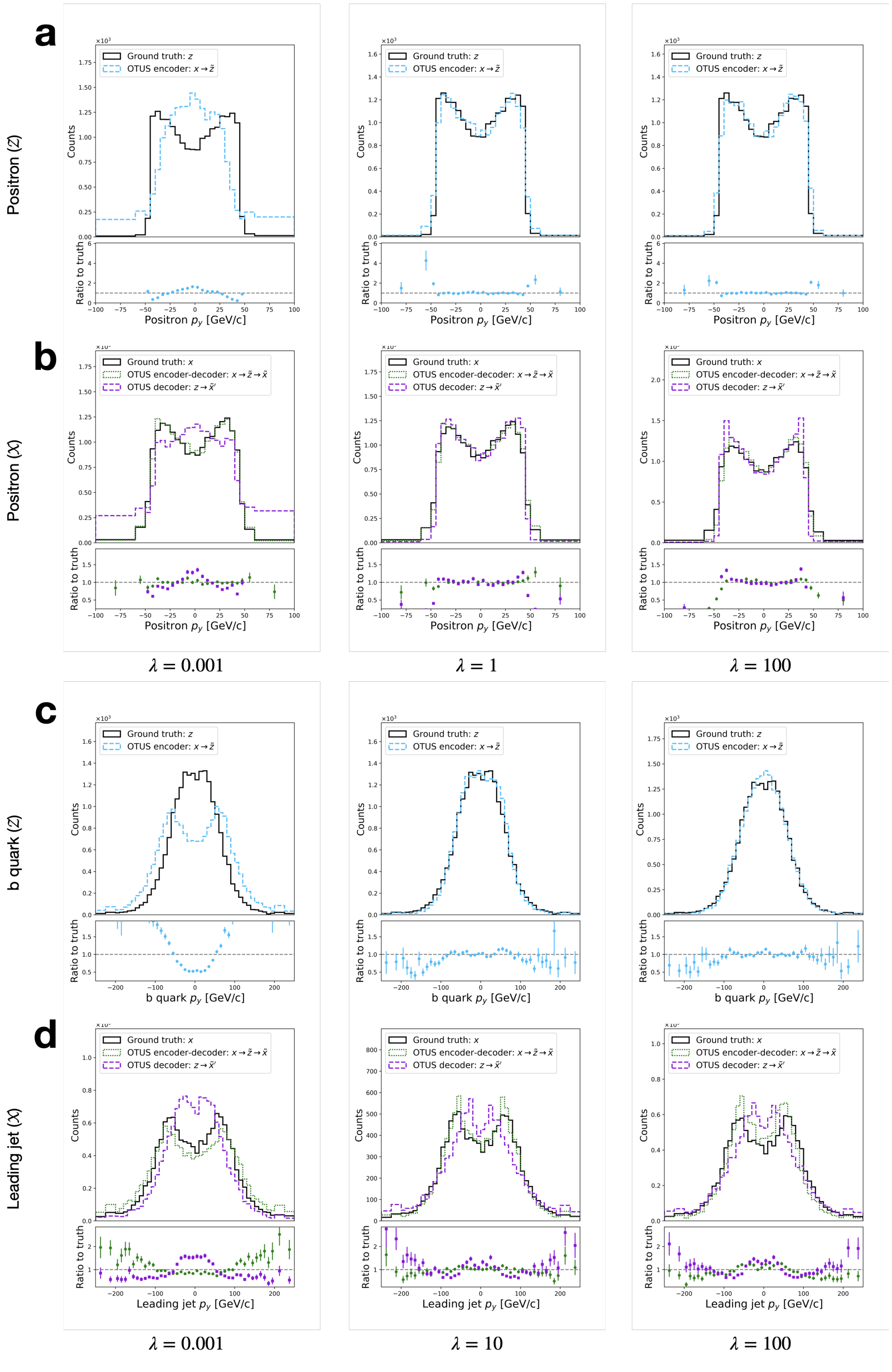

Supplementary Figure 3. Results of λ ablation study on principal axis matching. **a** Matching of the positron's p_y distribution for $\lambda = 0.001$, $\lambda = 1$, and $\lambda = 100$ in $\mathcal{Z}$ for the $Z \to e^+e^-$ study. **b** Matching of the positron's p_y distribution for $\lambda = 0.001$, $\lambda = 1$, and $\lambda = 100$ in $\mathcal{X}$ for the $Z \to e^+e^-$ study. **c** Matching of the b quark's p_y distribution for $\lambda = 0.001$, $\lambda = 10$, and $\lambda = 100$ in $\mathcal{Z}$ for the semileptonic $t\bar{t}$ study. **d** Matching of the leading jet's p_y distribution for $\lambda = 0.001$, $\lambda = 10$, and $\lambda = 100$ in $\mathcal{X}$ for the semileptonic $t\bar{t}$ study. For small values of λ ($\lambda = 0.001$) we find that performance suffers as latent space matching is not enforced. This improves as we increase λ but eventually plateaus.

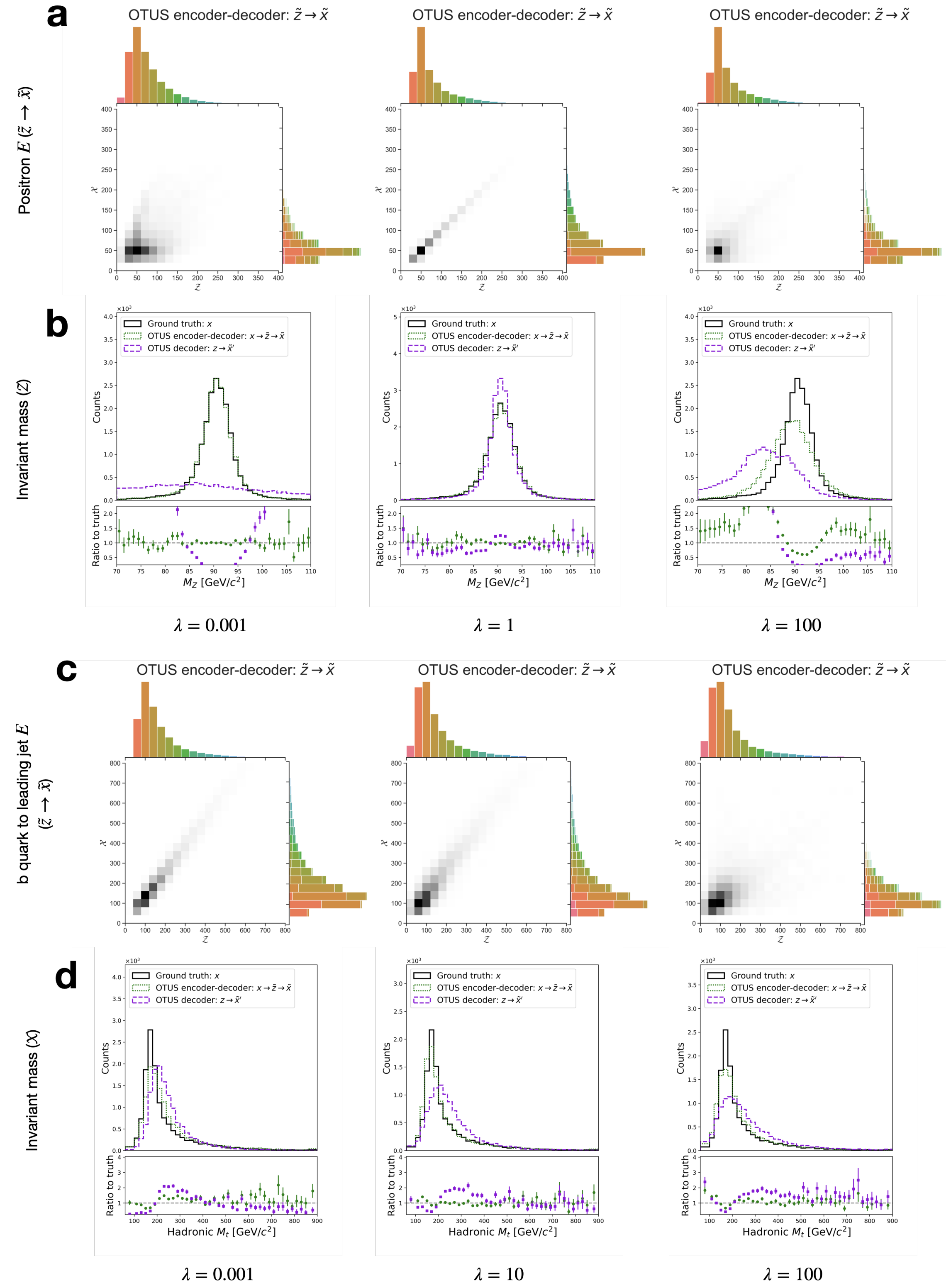

Supplementary Figure 4. Results of λ ablation study on transport plots and derived quantity matching. a Transport plans from $\tilde{z} \to \tilde{x}$ of the positron's E distribution for $\lambda = 0.001$, $\lambda = 1$, and $\lambda = 100$ for the $Z \to e^+e^-$ study. **b** Matching of the invariant mass of the Z-boson for $\lambda = 0.001$, $\lambda = 1$, and $\lambda = 100$ for the $Z \to e^+e^-$ study. **c** Transport plans from $\tilde{z} \to \tilde{x}$ of the b quark's E distribution in $\mathcal{Z}$ to the leading jet's E distribution in $\mathcal{X}$ for $\lambda = 0.001$, $\lambda = 10$, and $\lambda = 100$ for the semileptonic $t\bar{t}$ study. **d** Matching of the invariant mass of the top-quark, M_t, reconstructed using information from the hadronically decaying W-boson for $\lambda = 0.001$, $\lambda = 10$, and $\lambda = 100$ for the semileptonic $t\bar{t}$ study.

We see performance in matching principal axes improve as λ grows larger, possibly plateauing in the case of the semileptonic $t\bar{t}$ study. This plateau is potentially due to issues with optimization and poor numerical conditioning with overly large λ. However, we find that too large of a value of λ results in unphysical mappings.

Specifically, we find unphysical behavior when we view the transport plots and derived quantities (see Fig 4). Again, we note that this is less noticeable for the semileptonic $t\bar{t}$ study due to the ResNet [1] architecture. We find that the ideal choice is $\lambda \approx 1$ for the $Z \to e^+e^-$ study and $\lambda \approx 20$ for the semileptonic $t\bar{t}$ study; this retains acceptable principal axis matching while not introducing unphysical transformation characteristics. We suspect that if the choice of λ is too large, it over-constrains the optimization problem and should instead be annealed.

As discussed, instead of an expensive double-loop procedure where we train the encoder to optimality with the penalty method before updating the decoder, we forego theoretical considerations by jointly optimizing the encoder and decoder of a WAE on a surrogate loss as in [2]. The resulting loss is neither an upper nor a lower bound on the ideal objective $W_c(p(x), p_D(x))$, and we choose λ by experimentation. An alternative would be to use the Sinkhorn Autoencoder [4] approach, which only needs a large enough λ for its loss to be a proper upper bound on $W_c(p(x), p_D(x))$. This is further motivation that this method should be explored in future work.

References

1. He, K., Zhang, X., Ren, S. & Sun, J. Deep residual learning for image recognition. Preprint at https://arxiv.org/abs/1512.03385 (2015).

2. Tolstikhin, I., Bousquet, O., Gelly, S. & Schoelkopf, B. Wasserstein auto-encoders. Preprint at https://arxiv.org/abs/1711.01558 (2017).

3. Nocedal, J. & Wright, S. Numerical Optimization Second Edition (Springer, New York, 2006).

4. Patrini, G., et al. *Sinkhorn AutoEncoders* Preprint at https://arxiv.org/abs/1810.01118 (2018).
